# Draft Genome Sequence of the Beneficial Rhizobacterium Pseudomonas fluorescens DSM 8569, a Natural Isolate of Oilseed Rape (Brassica napus)

**DOI:** 10.1128/genomeA.00137-15

**Published:** 2015-03-26

**Authors:** Kai Nesemann, Susanna A. Braus-Stromeyer, Andrea Thuermer, Rolf Daniel, Gerhard H. Braus

**Affiliations:** a Department of Molecular Microbiology and Genetics, Institute of Microbiology and Genetics, Georg-August Universität, Göttingen, Germany; b Department of Genomic and Applied Microbiology & Göttingen Genomics Laboratory, Institute of Microbiology and Genetics, Georg-August Universität, Göttingen, Germany

## Abstract

Pseudomonas fluorescens DSM 8569 represents a natural isolate of the rhizosphere of oilseed rape (Brassica napus) in Germany and possesses antagonistic potential toward the fungal pathogen Verticillium. We report here the draft genome sequence of strain DSM 8569, which comprises 5,914 protein-coding sequences.

## GENOME ANNOUNCEMENT

Pseudomonas represents an abundant bacterial genus in many antagonistic root-associated communities (1). Pseudomonas fluorescens DSM 8569 was isolated from the rhizosphere of the *Verticillium* host oilseed rape (Brassica napus) in Rostock, Germany (2). The phytopathogenic fungus *Verticillium* requires an activator of adhesion for systemic infection of plant roots (3). The bacterium revealed a strong antimycotic effect on the phytopathogenic fungus *Verticillium* (4). A variety of secreted secondary metabolites with antimycotic impact were described in fluorescent pseudomonads, such as 2,4-diacetylphloroglucinol (DAPG), pyoluteorin, HCN, or pyrrolnitrin. A two-component system, *gacA*-*gacS*, was discovered in Pseudomonas protegens CHA0, which posttranscriptionally regulates the synthesis and secretion of these compounds (1). The synthetic pathway of phenazines in P. fluorescens 2-79 was investigated previously (5, 6). The chemical group of phenazines causes oxidative stress by accumulating toxic superoxide radicals and hydrogen peroxide in the target cell (7). Currently, it is unknown which suppressive mechanisms are responsible for the antagonistic potential of P. fluorescens DSM 8569. Genomic sequencing will be helpful in understanding the plant-promoting and antagonistic potentials of fluorescent pseudomonads.

The biocontrol strain P. fluorescens DSM 8569 was obtained from the DSMZ (Braunschweig, Germany). The genomic DNA was isolated using the MasterPure complete DNA and RNA purification kit (Epicentre, Madison, WI, USA). A shotgun sequencing library was generated, employing the Nextera DNA sample preparation kit, according to the manufacturer’s instructions. The whole genome of DSM 8569 was sequenced with the Genome Analyzer IIx (Illumina, San Diego, CA, USA). In total, 8.3 million paired-end reads of 112 bp were generated. The *de novo* assembly of all shotgun reads using SPAdes 3.0.0 (8) resulted in 135 contigs >3 kb and 119-fold coverage. The draft genome sequence comprises 6.6 Mb and a G+C content of 61.01%. Genome annotation was performed by the use of Prokka (9). The draft genome was found to harbor 2 rRNA clusters, 43 tRNA genes, 4,560 protein-coding genes with a predicted function, and 1,354 genes coding for hypothetical proteins.

The proteins involved in secondary metabolism were analyzed. The genes necessary for pyoluteorin synthesis (GenBank accession numbers 15560761, 15560764, 15560758, 15560774, and 15560768) and the entire phenazine operon described for P. fluorescens 2-79 (L48616.1) are absent. At least one gene (*phlG* [15563823]) required for the regulation of 2,4-diacetylphloroglucinol synthesis is missing in DSM 8569. In contrast, the genes responsible for HCN synthesis (15560558 and 15559866) are present in DSM 8569.

### Nucleotide sequence accession numbers.

This whole-genome shotgun project has been deposited at DDBJ/EMBL/GenBank under the accession no. JXOE00000000. The version described in this paper is the first version, JXOE01000000.

## References

[1] Haas D, Défago G 2005 Biological control of soil-borne pathogens by fluorescent pseudomonads. Nat Rev Microbiol 3:307–319. doi:10.1038/nrmicro1129. 15759041

[2] Berg G, Ballin G 1994 Bacterial antagonists to *Verticillium dahliae* Kleb. J Phytopathol 141:99–110. doi:10.1111/j.1439-0434.1994.tb01449.x.

[3] Tran VT, Braus-Stromeyer SA, Kusch H, Reusche M, Kaever A, Kühn A, Valerius O, Landesfeind M, Aßhauer K, Tech M, Hoff K, Pena-Centeno T, Stanke M, Lipka V, Braus GH 2014 *Verticillium* transcription activator of adhesion Vta2 suppresses microsclerotia formation and is required for systemic infection of plant roots. New Phytol 202:565–581. doi:10.1111/nph.12671. 24433459

[4] Berg G, Opelt K, Zachow C, Lottmann J, Götz M, Costa R, Smalla K 2006 The rhizosphere effect on bacteria antagonistic towards the pathogenic fungus *Verticillium* differs depending on plant species and site. FEMS Microbiol Ecol 56:250–261. doi:10.1111/j.1574-6941.2005.00025.x. 16629754

[5] Mavrodi DV, Ksenzenko VN, Bonsall RF, Cook RJ, Boronin AM, Thomashow LS 1998 A seven-gene locus for synthesis of phenazine-1-carboxylic acid by *Pseudomonas fluorescens* 2-79. J Bacteriol 180:2541–2548. 9573209 10.1128/jb.180.9.2541-2548.1998 PMC107199

[6] Mavrodi DV, Peever TL, Mavrodi OV, Parejko JA, Raaijmakers JM, Lemanceau P, Mazurier S, Heide L, Blankenfeldt W, Weller DM, Thomashow LS 2010 Diversity and evolution of the phenazine biosynthesis pathway. Appl Environ Microbiol 76:866–879. doi:10.1128/AEM.02009-09. 20008172 PMC2813009

[7] Hassett DJ, Woodruff WA, Wozniak DJ, Vasil ML, Cohen MS, Ohman DE 1993 Cloning and characterization of the *Pseudomonas aeruginosa sodA* and *sodB* genes encoding manganese- and iron-cofactored superoxide dismutase: demonstration of increased manganese superoxide dismutase activity in alginate-producing bacteria. J Bacteriol 175:7658–7665. 8244935 10.1128/jb.175.23.7658-7665.1993 PMC206923

[8] Bankevich A, Nurk S, Antipov D, Gurevich AA, Dvorkin M, Kulikov AS, Lesin VM, Nikolenko SI, Pham S, Prjibelski AD, Pyshkin AV, Sirotkin AV, Vyahhi N, Tesler G, Alekseyev MA, Pevzner PA 2012 SPAdes: a new genome assembly algorithm and its applications to single-cell sequencing. J Comput Biol 19:455–477. doi:10.1089/cmb.2012.0021. 22506599 PMC3342519

[9] Seemann T 2014 Prokka: rapid prokaryotic genome annotation. Bioinformatics 30:2068–2069. doi:10.1093/bioinformatics/btu153. 24642063

